# Learning medical professionalism – the application of appreciative inquiry and social media

**DOI:** 10.1080/10872981.2019.1586507

**Published:** 2019-03-04

**Authors:** Jyh-Gang Hsieh, Li-Chuan Kuo, Ying-Wei Wang

**Affiliations:** a Department of Family Medicine, Hualien Tzu Chi Hospital, Hualien, Taiwan; b Department of Medical Humanities, School of Medicine, Tzu Chi University, Hualien, Taiwan

**Keywords:** Medical professionalism, social media, appreciative inquiry, culture

## Abstract

**Background**: Medical professionalism is often considered difficult to be clearly observed and learned. However, although most medical students or residents affirm the necessity of medical professionalism courses, few agree that those currently offered are adequate for a medical career.

**Objective**: To develop a curriculum for teaching professionalism by enabling students to share positive examples of professionalism in social media that reflects the authentic experience in clinical environment.

**Design**: Between October 2015 and June 2017, the authors developed a clerkship program to teach professionalism with the support of social media and appreciative inquiry. Medical students were required to write posts on the positive behaviors they observed during clinical practice in the Facebook group. Other students and course instructors commented or responded to the posted content. The content on Facebook analyzed by course instructors and was based on the definition of medical professionalism according to the 18 attributes proposed by Cruess et al.

**Results**: In total, 103 medical students in their first clinical year participated and posted 435 records of role model learning in the Facebook group. The majority of students learned the most when the clinical instructors were passionate about their teaching and guidance in medical expertise; this accounted for 23.0% of all role model behaviors. Other attributes of professionalism that students appreciated most were being caring and compassionate (17.2%), competence (9.6%), openness (8.8%), and presence (7.7%). More than 90% of the students reported enjoying this type of course and would like to integrate their learning experiences into future behavior.

**Conclusions**: This innovative training program was well accepted in the formal curriculum and the predesigned social media environment. Appreciative inquiry for medical professionalism should be integrated into the organizational culture and the culture of social media interaction.

## Introduction

Medical professionalism has long been considered the basis for physicians to enter the medical profession and execute medical practices. However, even now, medical professionalism is regarded as part of medical education, which is difficult to concretize [1]. In fact, medical professionalism is often considered a theoretical construct that focuses on describing abstract qualities, not behaviors that can be clearly observed and learned. Although such qualities are clearly defined, they are not easily transformed into tangible and measurable learning outcomes [1,2].

Despite that almost all medical schools currently have formal courses in medical professionalism, most offer this type of course in the junior years, with only a few exceptions where they are offered in the clinical years [3,4]. Some schools include presentations on medical professionalism in white coat ceremonies, while others offer courses on various subjects including the discourse on literature and narrative medicine, history of medicine, discourse on clinical cases, and drama appreciation and performance. Such courses are distributed among different curriculums such as medical ethics or medical humanities courses [3]. However, despite that most medical students or residents affirm the necessity of medical professionalism courses, few agree that those currently offered are adequate for a medical career [5]. According to the guidelines for evidence-based medicine education issued by the Association for Medical Education in Europe (AMEE), the most effective technique to strengthen medical professionalism teaching is to integrate it into clinical medicine curriculums and conduct situated learning by allowing students to observe the behaviors of a role model. This guides them to perform critical reflection and gain a desirable and positive influence and ultimately internalize such behaviors into their own standards of conduct [1,4]. Irby et al. summarized past discussions on the development of medical professionalism into three major frameworks: virtue-based professionalism, behavior-based professionalism, and professional identity formation [6]. They suggested that pedagogy includes direct instruction, role models, case studies, reflective writing, guided discussions, appreciative inquiry, reflection on action, and so on. Thus, teaching professionalism should adopt a diversified approach.

The use of social media in daily life has now become a popular group culture among medical students, with more than 94% of those in America having a Facebook account through which they share medical and non-medical information with their peers [7]. As social media are widely used and accepted, many education professionals contemplate how to employ this trend to enhance students learning motivation and effectiveness. As such, numerous applied teaching programs supported by social media have been developed and desirable results achieved. The instant messaging functions of social media can create virtual office hours for instructors as an alternative to face-to-face discussions [8]. Furthermore, students can use social media to communicate and collaborate with their peers for learning purposes [9,10], enhancing their sense of participation and strengthening their personal reflections [11]. Appreciative inquiry emphasizes the discovery of positive merits to reinforce positive behavior, enhancing overall performance and transforming organizational culture [12]. It also allows medical students to learn about the behavior and values of medical professionalism in real situations and reflect on their understanding thereof by observing role model behaviors. This effective technique can be applied to medical professionalism teaching. This study utilized social media as a learning platform for professionalism and adopted appreciative inquiry and role model learning in professionalism teaching to promote students’ reflection on positive behaviors and establish an effective teaching tool. The results are used to analyze students’ knowledge of medical professionalism.

## Materials and methods

**Figure 1.**
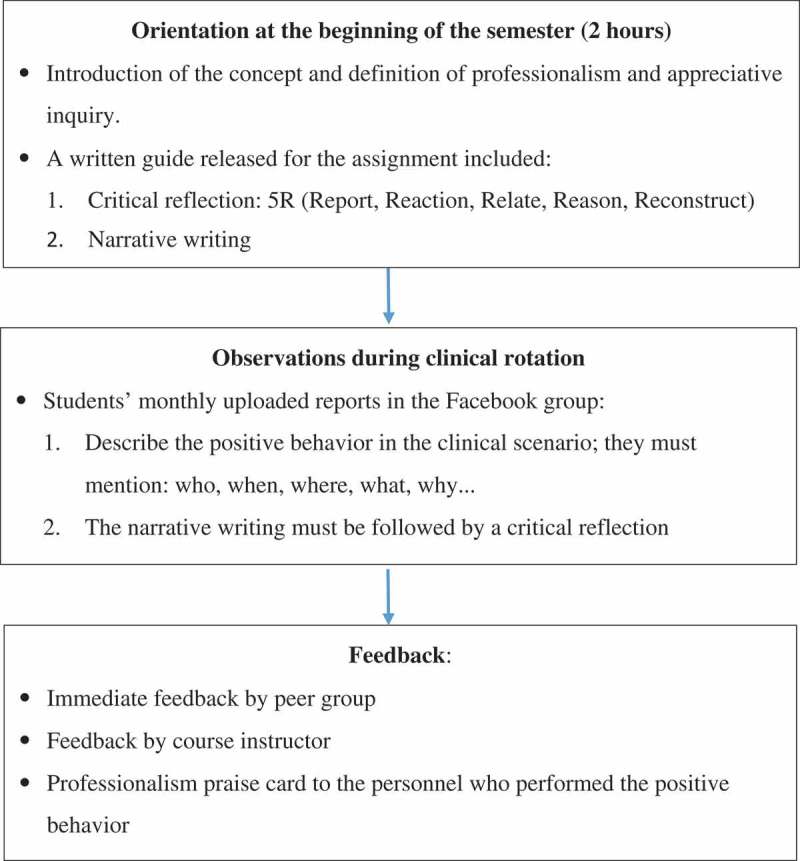
Curriculum procedures and requirements.

The School of Medicine, Tzu Chi University developed a course combined the existing elements of professionalism teaching in clinical practice and required credit courses on clinical communication and ethics and taught professionalism with the support of social media and appreciative inquiry from October 2015 to June 2017. It is a required curriculum for medical students in the last two years of their clinical rotation. Before the course started, a closed group was created on Facebook that only students and instructors involved in the course could join and browse or post comments. During the first two-hour lecture at the beginning of the semester, the course instructor explained the learning objectives to the participating students, provided guidance, and clarified concepts. Furthermore, the instructor introduced medical professionalism, role model learning, appreciative inquiry, and how social media was to be used in the course, and announced the submission regulations and other important issues (Figure 1).

The orientation course introduced the definition of medical professionalism to students using the 18 attributes proposed by Cruess et al., which are classified into three groups: attributes of the healer, attributes of the professional, and attributes of both the healer and professional [13]. The appreciative inquiry section applied the 4-D model (discover, dream, design, and destiny). Students were encouraged to share their experiences through dialogs and discuss the core issues they observed (discover), describe their thoughts and prospects on issues and build ideal conditions together (dream), and integrate different types of information and adjust their self-role (design) to construct ideal conditions in reality through continuous revision (destiny) [14,15]. The content posted by students on Facebook had to include self-reflections, and instructors explained and demonstrated how to apply critical reflection. Students were required to adopt the 5-R framework (report, reaction, relate, reason, and reconstruct) in their self-reflections, which helped them quickly connect and accumulate their new experiences with prior ones. The purpose was to establish medical professionalism among medical students through role model learning [16].

The course was integrated with students’ clinical practice. All participating students were required to write posts on the positive behaviors they observed during clinical practice on the appreciative inquiry Facebook group at least once a month. Furthermore, they were required to participate in or comment on discussions with other students. The assignments had to describe the people and relevant events students believed are worth learning about during the clerkship, and they had to mention the specific person, time, place, actions, and why such behavior should be regarded as model behavior. The people described were not limited to any type of medical personnel. Through their descriptions, we could make sense of the narratives students posted and understand the focus and values they pay attention to during clinical practice. When students commented or responded to the published content of other students, they needed to write their thoughts on the circumstances or events others described, as well as what these events meant to them.

The content feedback was divided into three parts. The first part was peer feedback from other students on Facebook in response to personal experiences and feelings, which is aligned to the essence of appreciative inquiry. The second was feedback provided by course instructors on the content posted by students to guide their reflection process and enable them to integrate their learning experiences into future behavior. Three faculties from the department of medical humanities participated as course instructors. They had to keep reminding students of the purposes and requirements of the curriculum and emphasize the narration of positive cases. If a remark or symbol in a student post could be used to identify a patient, instructors acted as moderators to avoid the disclosure of the private details of any individual. The third part was in the form of a specifically named professionalism praise card, which was regularly sent during the semester by the lead instructor to personnel whom the students referred to as role models in their posts. Since the praise cards were sent to the clinical role models after students finished their clinical rotation of that department, there was no relationship between the praise cards and the students’ grades. Praise cards reported the actions students observed and their impact. This process was also considered as appreciative inquiry toward the observed personnel. Clinicians could also reflect on their actions and connect with their experiences to analyze the significance of such behavior in the medical field and the inspiration it could offer students. Actively engaging in self-adjustment and re-constructing similar events in the future can achieve positive reinforcement among clinical instructors and be an effective way of teacher development.

## Results

After obtaining approval from the Ethics Committee, the course began collecting statistics for a two-year period from October 2015 to June 2017. In total, 103 medical students in their first clinical year participated, including 50 males (48.5) and 53 females (51.5%). The age range was 21–30 years. The largest group was aged 22 years, with 58 participants (56.3%), followed by those aged 23, with 23 participants (22.3%) (Table 1).

**Table 1.** Demographics of medical students who participated the curriculum.

	N = 103 N (%)
**Age (y)**	
≤21	8 (7.8%)
22	58 (56.3%)
23	23 (22.3%)
24	7 (6.8%)
≥25	7 (6.8%)
**Gender**	
Male	50 (48.5%)
Female	53 (51.5%)

Students published 435 records of role model learning in the Facebook group. The main personnel observed were attending physicians (78.5%), followed by residents (15.8%), nurses (3.8%), and other students (1.9%). The content on Facebook analyzed by course instructors and was based on the definition of medical professionalism according to the 18 attributes proposed by Cruess et al., which are classified into three groups: attributes of the healer, attributes of the professional, and attributes of both the healer and professional. Passion for teaching and communication skills were added as additional attributes, as they were frequently mentioned in students’ published content, but not included in the attributes outlined above. The results of the analysis showed that the majority of students believed they learned the most when the clinical instructors were passionate about their teaching and guidance in medical expertise, accounting for 23.0% of all role model behaviors. In addition, the professionalism students felt tended to be related to attributes of the healer, the most significant ones being caring and compassion (17.2%), openness (8.8%), and presence (7.7%). These were followed by attributes of both the healer and professional, the most significant being competence (9.6%) and altruism (5.7%). In terms of attributes of the professional, the most significant were responsibility to the profession (7.6%) and responsibility to society (4.2%) (Table 2).

**Table 2.** Analysis of major attributes of medical professionalism in Facebook.

Attributes of Medical Professionalism	Proportion of Mentions (%)
**Passionate about teaching and guidance in medical expertise**	23.0
**Attributes of the healer**	
Compassion	17.2
Openness	8.8
Presence	7.7
**Attributes of the professional**	
Responsibility to the profession	7.6
Responsibility to society	4.2
**Attributes of both the healer and professional**	
Competence	9.6
Altruism	5.7

A student satisfaction survey was conducted after the course, wherein more than 90% of the students reported enjoying this type of course and would like to integrate their learning experiences into future behavior. They believed that exemplary behavior displayed by role models is worth learning and that they were able to attain professional growth in various aspects of the medical profession. The students expressed very few negative comments about the course. Only two students mentioned that during the course, they were only allowed to describe the positive behavior of instructors and considered it unnecessary to merely praise the instructors. In response to this comment, the instructors explained that appreciative inquiry was not only intended for instructors. The purpose of exemplifying positive behaviors within the community was that all students could understand, reflect on, and strengthen their medical professionalism.

## Discussion

The above analysis shows that there are evident cultural differences in medical professionalism. Certain traditions that are highly regarded in Western medicine are rarely mentioned in students’ work, including integrity and honesty, autonomy, insight, and respect for patient dignity and autonomy. On the contrary, traditional oriental medicine emphasizes that physicians should ‘treat patients like family,’ and the focus of students’ observations are the care and enthusiasm while wholeheartedly attending to and accompanying patients. In fact, such a finding begs the question: Can Western medical professionalism education be directly transferred to other cultural areas without causing conflict or being questioned [17]? From the perspective of medical students, our results confirm that cultural differences influence students’ professional identity and that medical professionalism education should also be adjusted according to culture.

Moreover, it is important for us to rethink students’ emphasis on the enthusiasm of clinical instructors. In fact, in addition to professional status, another important role for clinical professionals could be as instructors of clinical students. Teachers must have a passion for teaching in order to construct a safe learning environment for students and promote effective learning among them. Enthusiastic teachers can provide students with sufficient support and meet the expectations of teachers and students in terms of teaching goals. As role models, this is also the promise that teachers should make to students and teaching [18]. From a cultural perspective, we find that modern Western medical education has only, for the past 20 years, begun to focus on the concept of mentoring [19], while traditional Chinese medical education has always been based on a system of masters and apprentices and emphasizes that ‘example is better than precept.’ Thus, in oriental culture, clinical instructors become role models for students. This is the reason we consider our planned program suitable for medical education in a Chinese cultural context.

Social media are common communication tools used by students in this modern era. This course used Facebook as a teaching aid to conduct professionalism teaching by adopting an appreciative inquiry model, and was considered a mandatory formal curriculum. The theoretical framework set out in Kirkpatrick’s model was applied to understand the changes to individual students, clinical instructors, and organizational culture after offering this course. We also observed whether participating students could describe positive behaviors observed during clinical practice using social media in daily life after the course. This naturally transformed the formal curriculum into a hidden curriculum, which continued to impact students. Furthermore, we observed whether students displayed behaviors that reflected professionalism in their daily work.

Miller proposed a four-part pyramidal structure as a framework within which the multiple levels of mastery over the art and science of medicine could be assessed [20]. The real objective of teaching professionalism is to assist students as they develop their own professional identities. It is proposed that a fifth level ‘Is’ be added at the apex of the pyramid to reflect the presence of professional identity. As learners progress up the pyramid, for ‘Knows,’ learners would be expected to ‘Know the behavioral norms expected of a physician.’ The behavioral norms of medicine’s community of practice must be communicated explicitly to every learner. At the ‘Knows How’ level, it would be necessary to ‘Know when individual behaviors are appropriate [21].’ Critical elements of professional identity formation (PIF) included guided reflection, use of personal narratives, the integral role of relationships and role modeling [22].

The other focus of the study was for the clinical instructors and physicians being observed to reflect on and modify their teaching behavior by offering them feedback written on professionalism praise cards. Weissmann et al. found that self-awareness as a role model is a quality of good clinical instructors [23], while Steinert et al. emphasized the possible importance of role modeling in the reinforcement of teacher development [24]. Medical professionals are often unaware of the impact of their behavioral patterns on students. In fact, these role models not only play their part, but also make efforts in real life in acting out their functions, which motivates students to learn [25]. Therefore, the next step is for clinical instructors and students to participate together in social media and achieve the objective of direct interaction. We hope that role model learning will promote faculty development and strengthen professionalism teaching. Teaching, learning, and assessment are to be integrated into a professional attitude and beliefs to achieve the objective of the next phase.

## Conclusions

Role model learning is based on observing the interactions between clinical instructors and patient care, and it is a central process for developing professional identity among medical students [26]. It establishes an effective link between individuals and the medical system and strengthens the individual’s knowledge about behavioral norms and attitudes towards medical professionalism through the process of guided reflection. Our innovative training program was well accepted in the formal curriculum and the predesigned social media environment, and it also adapts to the needs of different cultures in medical professionalism education. Appreciative inquiry for medical professionalism should be integrated into the organizational culture and the culture of social media interaction. It is expected that students can continue daily discussion of positive role models on the social media platform. Faculty development and teaching that strengthens professionalism will also be promoted by the role-model learning curriculum in the next steps.
